# Antibody kinetics and clinical course of COVID-19 a prospective observational study

**DOI:** 10.1371/journal.pone.0248918

**Published:** 2021-03-22

**Authors:** Anna Bläckberg, Nils Fernström, Emma Sarbrant, Magnus Rasmussen, Torgny Sunnerhagen

**Affiliations:** 1 Division of Infection Medicine, Department of Clinical Sciences Lund, Lund University, Lund, Sweden; 2 Clinic of Infectious Diseases, Skåne University Hospital, Lund, Sweden; 3 Department of Clinical Microbiology, Office for Medical Services, Region Skåne, Lund, Sweden; Heidelberg University Hospital, GERMANY

## Abstract

**Background:**

Serological response and association to clinical manifestation is important for understanding the pathogenesis of COVID-19.

**Materials and methods:**

A prospective observational study was conducted where antibody responses of IgG and IgA towards SARS-CoV-2 spike protein were studied over time in patients with COVID-19. Possible associations between antibody titers and outcome were analyzed.

**Results:**

Forty patients with COVID-19, hospitalized at Skåne University hospital, Sweden, between April and June 2020 were included. IgG antibody responses were detected for all patients with the highest levels four weeks after COVID-19 diagnosis. Levels of IgA were generally higher at diagnosis and decreased towards baseline 4 weeks after confirmed COVID-19. Patients with severe COVID-19 had higher levels of antibodies directed against SARS-CoV-2 spike protein compared with patients with mild disease.

**Conclusion:**

IgG and IgA antibodies towards the spike protein follow different kinetics during COVID-19 and patients with severe disease develop higher antibody levels.

## Introduction

The Severe Acute Respiratory Syndrome Coronavirus 2 (SARS-CoV-2) has spread throughout the world causing a pandemic with coronavirus disease-19 (COVID-19). The majority of patients develop a mild respiratory or an asymptomatic infection while other patients acquire severe pneumonia with substantial respiratory support [1].

Coronaviruses are positive stranded RNA viruses of which the spike (S) glycoprotein and nucleocapsid (N) proteins are two important proteins responsible for cellular fusion and viral assembly respectively [2, 3].

Kinetics of antibody responses following COVID-19 has been described in several studies, comprising development of IgA, IgM and IgG against the N- and S protein [4–6]. Secretory IgA is an important defender of the mucosal surfaces and can neutralize SARS-CoV-2 by inhibiting the binding to the epithelial cells in the respiratory or digestive system [7]. Seroconversion of IgM to IgG typically occurs in the majority of patients with COVID-19 within 2–4 weeks from start of the infection, but time varies between debut of symptoms and seroconversion [6, 8–10]. Seow *et al* reported an average higher titer of neutralizing antibodies in patients with severe COVID-19, however no correlation of antibody kinetics was established [11]. Additionally, studies have reported lower titers of IgG in patients requiring ICU treatment care [12].

Previous studies have shown diverging results as to the differences in antibody responses in groups with different disease severity. This prospective observational cohort study was thus performed to provide new data with repeated antibody measurements correlated with clinical data and thus help fill this gap of knowledge.

## Materials and methods

### Patient cohort

Patients > 18 years of age with COVID-19 (confirmed by RT-PCR) and who were admitted to Skåne University Hospital in Sweden (which has a primary catchment area of approximately 370 000 as well as being a tertiary referral hospital) during the first wave of COVID-19 from late April to mid-July 2020 were included after oral and written consent. Patients who were admitted directly to the Intensive Care Unit (ICU) were excluded as they were unable to give informed consent. Serial blood samples were obtained day of inclusion (day 0), 3, 7, 10, 14 and on day 28. If patients were discharged during the study period, no blood samples were obtained until a follow up consultation on day 28.

Whole blood was collected in BD Vacutainer serum tubes and set to coagulate. Serum was prepared by centrifugation at 150 *g* for 10 minutes and stored at -80°C until use to investigate antibody responses through enzyme linked immunosorbent assay (ELISA) of IgG and IgA. Medical records were studied after a pre-specified protocol recording comorbidities such as cardiovascular disease, hypertension and respiratory disease. Medical treatment during hospital stay, imaging, need for treatment at an intermediate care unit or ICU, and outcome were also noted.

### ELISA

In order to investigate the kinetics of specific IgG and IgA against spike-protein of SARS-CoV-2, ELISA was used. Spike-protein (S1+S2 ECD, catalogue number 158-40589-V08B1-100, (NordicBiositer,Sino Biological) 0,02% was diluted in coating buffer and Nunc MaxiSorp (ThermoFisher Scientific) 96-well plates were coated. Incubation and washing steps were then performed as described in an earlier publication [13]. After another wash in PBST, two different mixtures were used either Protein G-Horse Radish Peroxidase (Bio-Rad Laboratories, USA) 0.03% in PBST to detect specific IgG or a Goat Anti Human-IgA-HRP (Bio-Rad Laboratories, USA) 0.02% in PBST to detect IgA. Further incubations and washing steps were done as described in the reference [13]. Results were analyzed in VICTOR Multilabel Plate Reader at 415 nm. All serum samples were analyzed in triplicate. To relate patient samples analyzed at different times, positive and negative reference samples were used on all plates. The positive reference was a convalescent serum sample taken from a patient with confirmed COVID-19 and which showed a strong IgG-band against spike protein (ZetaGene COVID-19 Rapid IgM IgG Test). The negative references convalescent serum from a patient with *Streptococcus dysgalactiae* bacteraemia, occurring in 2017.

### Ethics

The study protocol was subjected to ethical review by the Swedish ethical review authority, and the study was granted ethical approval (2020/01747).

### Statistics

Data was imputed in REDCap Software and later extracted to Microsoft Excel 2016 (Microsoft Corporation). Statistical analysis was performed in GraphPad Prism, version 8 (GraphPad Software). Fisher’s exact test was applied for categorical data and for comparison of continuous variables Mann-Whitney *U* test was performed. When comparing paired observations Wilcoxon matched-pairs signed rank test was applied. Significance was defined as a *p* values less than 0.05.

## Results

### Patient inclusion and grouping

Forty patients with COVID-19 who were admitted to the Clinic of Infectious Diseases between April and June 2020 were prospectively included in the study. Serial blood samplings were obtained on day 0 (*n* = 40), 3 (*n* = 25), 7 (*n* = 11), 10 (*n* = 6), 14 (*n* = 5) and on day 28 (*n* = 36). Patient characteristics are summarized in Table 1. The median age was 58 years (IQR 49–71) and a majority were men (55%). Twenty-nine patients had interstitial pneumonia and two patients experienced myocarditis and pulmonary embolism respectively. Five patients were nosocomially infected. The median number of days from onset of symptoms and COVID-19 diagnosis was 8 (IQR 7–11). The median length of stay was 6 days (IQR 4–10).

**Table 1:** Demographics and clinical patient characteristics.

Demographics	Study cohort	Group 1	Group 2	Group 3
	*n* = 40	*n* = 18	*n* = 16	*n* = 6
***Characteristic***				
Age, median	58 (49–71)	59 (52–64)	54 (48–69)	63 (46–83)
Sex, female *n* (%)	18 (45)	9 (50)	7 (44)	2 (33)
CCI, *n*	2 (1–5)	2 (1–4)	2 (0–4)	3 (1–7)
Hypertension *n* (%)	16 (40)	5 (28)	7 (44)	4 (67)
BMI, median	28 (24–32)	26 (22–30)	28 (24–34)	30 (26–34)
Nosocomial infection *n* (%)	5 (13)	4 (22)	0 (0)	1 (17)
Onset of symptoms to COVID-19 diagnosis (d), median	8 (7–11)	9 (8–20)	8 (6–11)	8 (5–8)
Days hospitalized, median	7 (5–11)	5 (3–10)	7 (5–10)	12 (9–33)
LMWH/NOAC/warfarin, *n* (%)	32 (80)	10 (56)	16 (100)	6 (100)
Antibiotics *n* (%)	23 (58)	5 (28)	12 (75)	6 (100)
Plasma *n* (%)	2 (5)	0 (0)	2 (13)	0
Steroids *n* (%)	8 (20)	4 (22)	2 (13)	2 (33)
Treatment at ICU or IME (*n*) (%)	3 (8)	0 (0)	0 (0)	3 (50)
In hospital mortality *n* (%)	2 (5)	0 (0)	0 (0)	2 (33)
***Clinical parameters***				
CRP (mg/L), median	92 (37–135)	43 (12–114)	117 (61–167)	125 (72–294)
WBC (10^9^ c/L), median	8 (5–12)	7 (4–10)	8 (5–11)	12 (8–17)
Neutrophils (10^9^ c/L), median	5 (4–9)	4 (2–7)	6 (4–9)	8 (4–14)
Lymphocytes (10^9^ c/L), median	1 (0.7–1.4)	1 (0.9–1.6)	1 (0.6–1.3)	0.8 (0.5–1)
Platelets (10^9^ /L), median	233 (185–299)	239 (175–315)	239 (196–314)	186 (138–272)
PCT (μg/L), median**	0.1 (0.07–0.3)	0.07 (0.05–0.1)	0.16 (0.1–0.3)	0.4 (0.1–1.2)
IL-6 (ng/L), median	57 (20–115)	24 (19–111)	58 (24–91)	124 (53–236)

Charlson Comorbidity score (CCI), body mass index (BMI), C-reactive protein (CRP), white blood cell (WBC), procalcitonin (PCT), low molecular weight heparin (LMWH), new oral anticoagulant (NOAC), intensive care unit (ICU), intermediate care (IME). For continuous variables interquartile range (IQR) is defined in the brackets. All clinical parameters are the median of every highest value for each patient, (except lymphocytes platelets where the lowest value for each patient was obtained.

* Defines statistical significance; CRP, (*p* < 0.05), between group 1 vs 2, adjusted *p* < 0.05.

** Defines statistical difference; PCT, (*p* < 0.001), between group 1 vs 2, adjusted *p* < 0.05 and group 1 vs group 3, adjusted *p* < 0.05.

Patients were separated into three different groups based on supportive respiratory treatment. Group 1 comprised patients with mild COVID-19 (*n* = 18), where oxygen treatment was not required, whereas group 2 consisted of patients with moderate COVID-19 (*n* = 16), requiring 1–6 liters per minute of supplementary oxygen. Patents in group 3 acquired severe COVID-19 (*n* = 6) involving non-invasive ventilation, high-flow nasal cannula oxygen therapy, ICU treatment and/or death. Two patients died during the study period prior to blood sampling on day 28. The medians of every highest value of every patient’s clinical parameters were compared within the groups, with exception of lymphocytes and platelets where the medians of every lowest value of every patient’s clinical parameters were obtained. There was a statistical significance in levels of CRP within the groups (*p* = 0.02), Kruskal-Wallis test, applying Dunn’s Multiple comparison test, such difference was observed between group 1 and 2, 43 vs 117 (adjusted *p* = 0.04). Utilizing Kruskal Wallis there was a statistical significance in levels of PCT within the groups, (*p* = 0.01), applying Dunn’s Multiple comparison test, such difference was observed between group 1 and 2, 0.007 vs 0.16 (adjusted *p* = 0.04) and between group 1 and 3, (adjusted *p* = 0.02).

### Kinetics of IgA and IgG from day 0 and day 28

**Fig 1:**
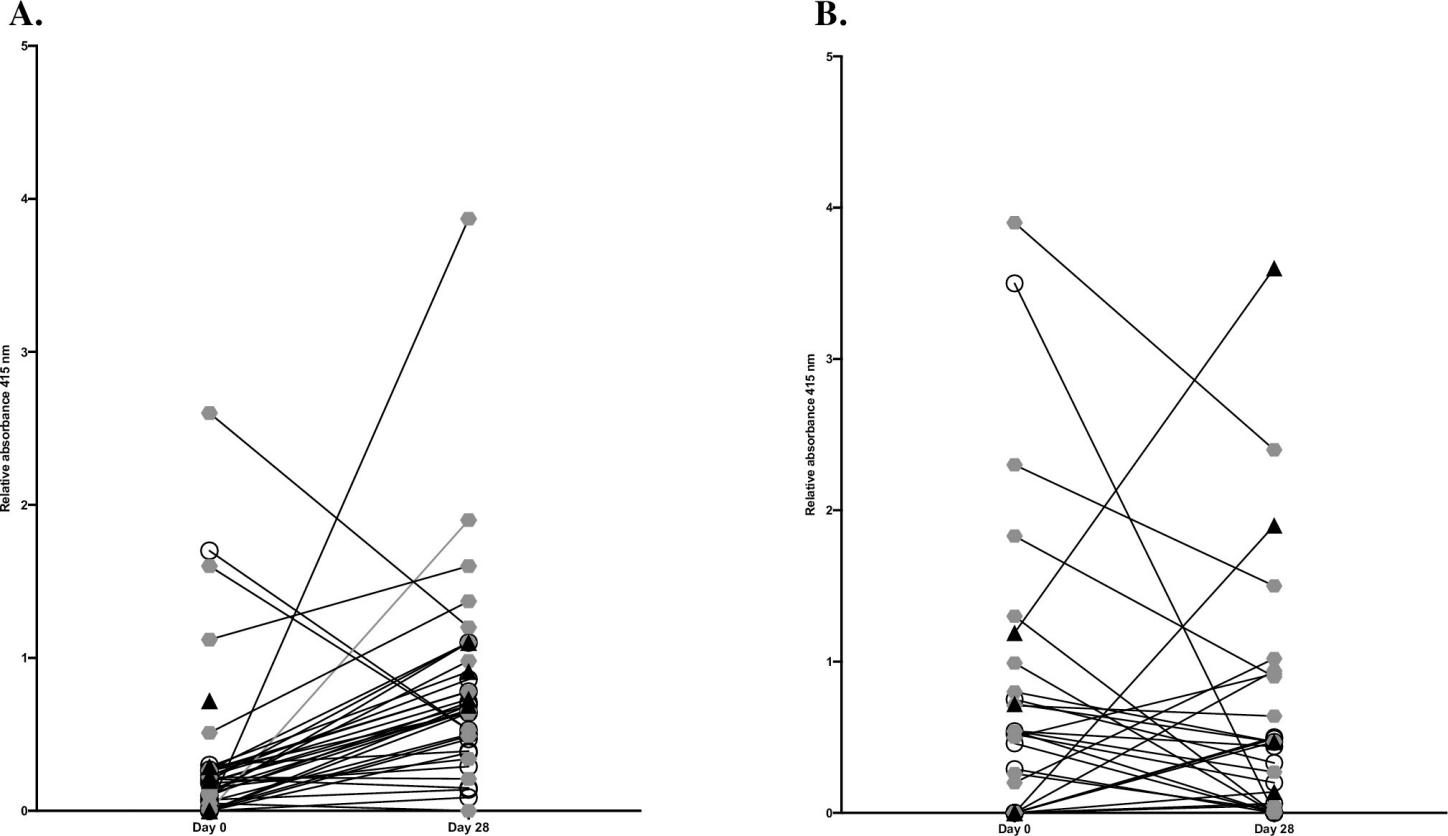
Relative levels of absorbance from day 0 to day 28. Relative levels of absorbance of IgG (1A) and IgA (1B) against SARS-CoV-2, separated on the three study groups were compared from day 0 and day 28. Group 1 represent patients with mild disease, group 2 include patients with moderate disease and group 3 encompass patients with severe COVID-19. White circle indicates group 1, grey hexagon indicates group 2 whereas black triangle defines group 3.

Firstly, the serological response towards SARS-CoV-2 spike protein of the study cohort was investigated looking at development of IgG and IgA from day 0 to day 28 (Fig 1). There was a statistically significantly increase in median levels of relative absorbance for IgG between day 0 and day 28, 0.19 vs 0.66, Wilcoxon matched-pairs signed rank test, (*p* = 0.0002) (Fig 1B). A propensity of decreased median levels of relative absorbance for IgA between day 0 and day 28 was observed and was not statistically significant, 0.3 vs 0.2, Wilcoxon matched-pairs signed rank test showing *p* = 0.4) (Fig 1A).

**Fig 2:**
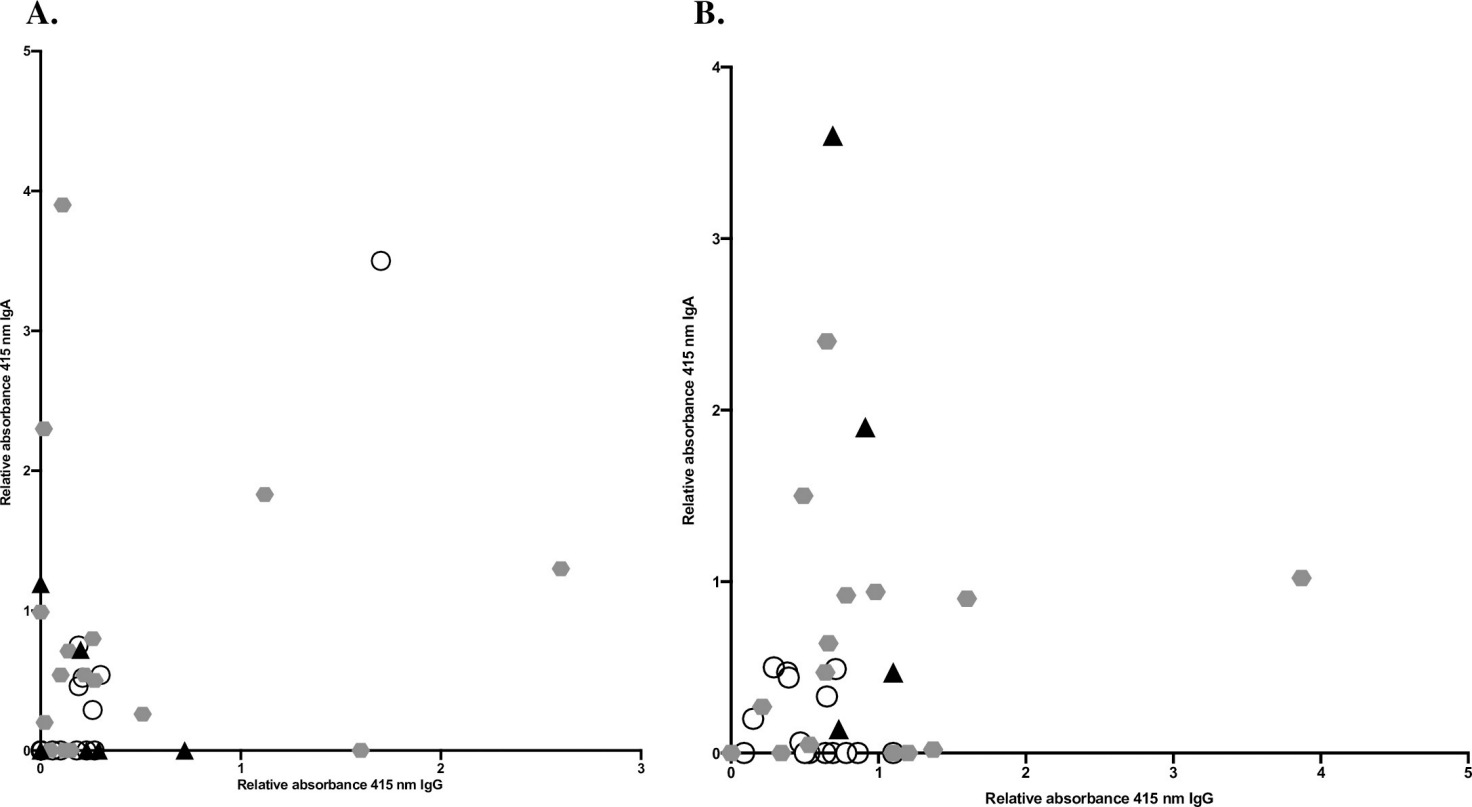
Correlation of relative levels of absorbance of IgG and IgA. Relative levels of absorbance of IgG in correlation to IgA day 0 (2A) and day 28 (2B), IgA day 0 (2C) and IgA day 28 (2D) were compared. All values were differentiated based on disease severity.

There was no correlation in levels of absorbance of IgG and IgA on day 0, Spearman correlation 0.2, *p* = 0.2, (Fig 2A) or on day 28, Spearman correlation 0.2, *p* = 0.4, (Fig 2B).

Five patients in this study had levels of IgG (range 29–100%) that decreased from day 0 to day 28. When comparing these five patients with decreasing levels of IgG to patients with increasing IgG levels, (*n* = 29), the clinical characteristics were similar (S1 Table). One patient with decreasing IgG levels received cortisone whereas three patients in the group with increasing levels received cortisone and one received plasma products. Two patients died prior to blood sampling on day 28. Both patients showed decreasing levels of IgG prior to death. They had interstitial pneumonia, high flow nasal cannula therapy and one of them received cortisone.

### Difference in median levels of IgG and IgA and disease severity

**Fig 3:**
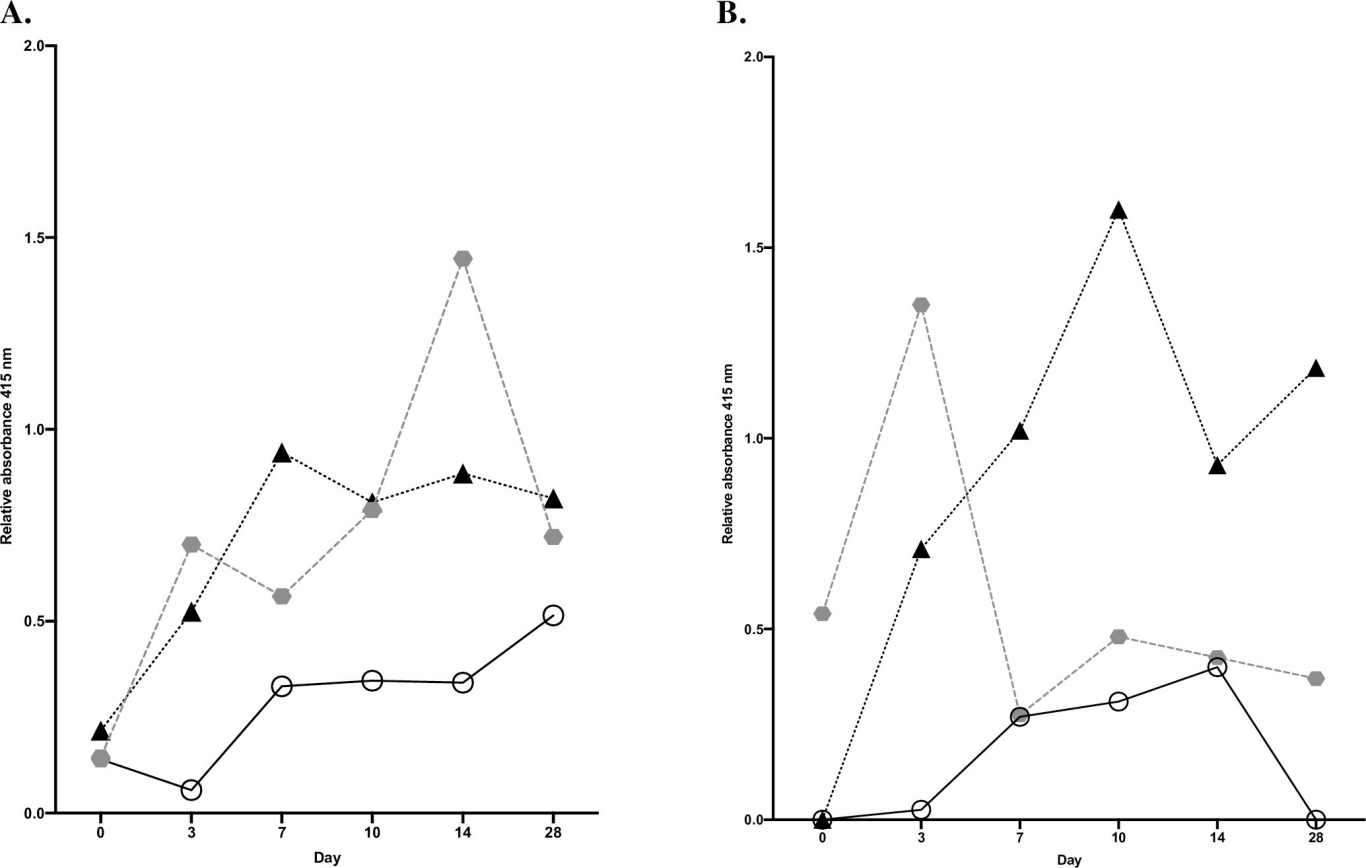
Median relative levels of absorbance during COVID-19. The medians of relative levels of absorbance of IgG within the groups based on every median levels, day 0–28 for IgG (3A) and IgA (3B) were compared.

Every median levels of relative absorbance for each group on day 0, 3, 7, 10, 14 and day 28 was calculated and compared together. The medians for each time point were compared within the groups, (Fig 3A and 3B). We then observed a statistical significant difference of median levels of IgG, Kruskal-Wallis test, *p* = 0.02, using Dunn’s Multiple comparison test, such difference was observed between group 1 and 3, 0.34 vs 0.82 (*p* = 0.04), (Fig 3A). Applying the same analysis for IgA, there was a statistically significant difference in median levels of IgA within the groups, Kruskal-Wallis test, *p* = 0.02, using Dunn’s Multiple comparison test, such difference was observed between group 1 and 3, 0.15 vs 0.98, *p* = 0.02, (Fig 3B).

We also analyzed potential differences on each time point separately by analyzing all values in every group and make comparison. There was a statistical difference in median levels of IgG within the groups on day 3, Kruskal-Wallis test, *p* = 0.02, using Dunn’s Multiple comparison test, such difference was observed between group 1 and 2, 0.06 vs 0.7, *p* = 0.02. When it comes to levels of IgA there was no statistical difference in levels of IgA within the groups on all serial days of blood samplings.

### Evolution of levels of IgG or IgA between day 28 and day 0 and disease severity

Additionally, intra-group development of antibodies was studied from day 0 and day 28 for IgG and IgA respectively and compared within the three groups. This analyses was to investigate evolution of antibodies between only day 0 and day 28, separated by disease severity. There was no statistical difference in the development of levels of IgG, (*p* = 0.1) or IgA, (*p* = 0.09) and disease severity, Kruskal-Wallis test.

### Levels of IgA and IgG and onset of symptoms for group 3

**Fig 4:**
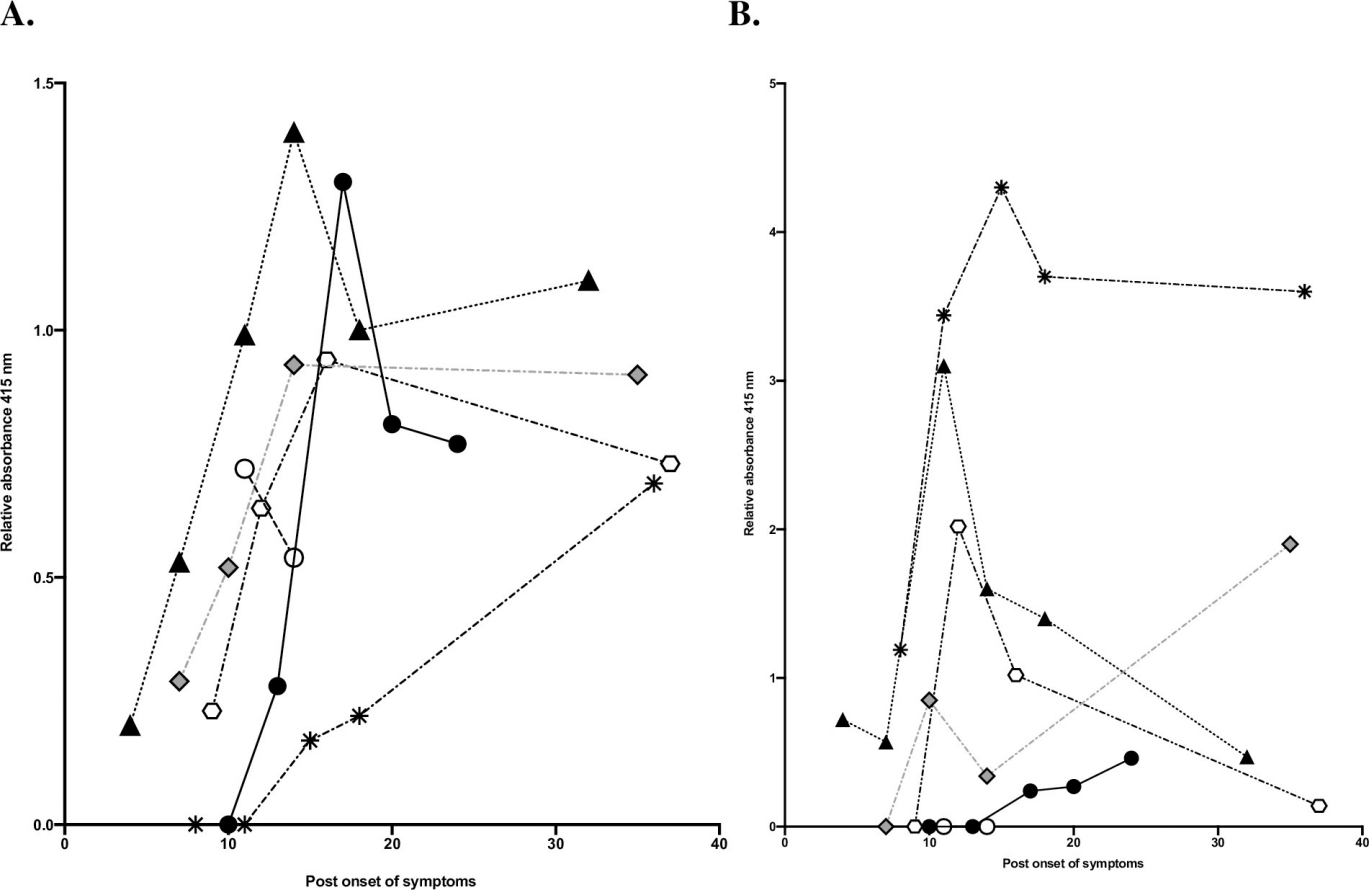
Relative levels of absorbance and post onset of symptoms. Relative levels of absorbance for IgG (4A) and for IgA (4B) for each patient in group 3, and relation to post onset of symptoms were compared. Each color and symbol shape represents a patient, black and white circles represent values from the two patients who deceased during the study period. Triangle indicates values from the one patient who required treatment at ICU.

To study the kinetics of antibodies in severe disease we focused on group 3. Levels of IgG (Fig 4A) and IgA (Fig 4B) for this group are shown in relation to post onset of symptoms. IgG levels are highest at day 15 post onset of symptoms and then slightly decreasing until day 38. Levels of IgA were on average highest on day 10, decreasing towards the baseline at day 38.

## Discussion

Our study provides insight in the course of COVID-19 and in the antibody kinetics for hospitalized patients. The included cohort was limited by the fact that patients who were admitted directly to the ICU were not possible to include, and the fact that the catchment area for Skåne University Hospital was relatively spared from the COVID-19 pandemic during the spring and summer of 2020. Infection was defined as having detectable levels of viral RNA in nasopharynx as measured by RT- PCR, and no quantification of viral load was made. Later studies have shown that detection of SARS-CoV-2 in serum is associated with worse prognosis, and the lack of such analysis is a limitation of the current study [14].

The choice to study IgG and IgA against SARS-CoV-2 spike protein was made both due to practical concerns regarding the availability of expressed viral proteins, and the fact that there was a hope that there would be a correlation between patients primarily isotype switching to one or the other antibody type and clinical characteristics. A link between humoral IgA and clearance of virus in early COVID-19 has been found, indicating the importance of IgA [15]. No such clear association between clinical presentation and isotype was found in this study, possibly due to the sample size.

Our study showed a development of IgG and IgA towards S protein from inclusion to follow up 28 days later, with more severely ill patients tending towards having higher IgG titers. This is in line with other studies, and another study from Sweden demonstrated higher levels of anti-SARS-CoV-2 antibodies in patients with severe COVID-19 compared to patients with a milder presentation [16].

Fifteen patients had levels of IgA at least 20% lower at day 28 than at day 0 (range 35–100%, with 5 patients having measurable levels of IgA at inclusion but not day 28), the majority of which had moderate COVID-19 (group 2), *n* = 9. Three of them also showed a similar decrease in levels of IgG. One patient with declining levels of IgA suffered from severe COVID-19 (group 3) and the rest presented with a mild infection (*n* = 5). Several patients had very low levels of IgA which might be due to the fact that IgA was measured in serum and not in saliva. However, significant levels of humoral IgA directed against SARS-CoV-2 spike proteins have been found in other settings such as in the study by Sterlin *et al* [15]. Nevertheless, the results of this study are in line with previous results in that the levels of IgA seem to decline at day 28 as compared to earlier in the course of disease [15].

Analyzing the results of this study it is clear that the levels of antibody responses vary greatly between patients (even when they had a similarly severe disease), confirming earlier studies. This information should inform further studies on COVID-19 infection and antibody levels, as it means that the sample size needs to be larger to account for this variation.

Antibody responses following covid-19 play a part in disease progression where intravenous immunoglobulin and monoclonal antibodies may be a therapy of choice in certain patients [17–19]. We chose to look at one protein, the S1 and S2 domains of S protein to establish antibody responses. Currently several COVID-19 vaccines directed against the S protein are developed, manufactured and also administered [20–23].

This study does not provide information regarding the length of antibody duration beyond 28 days, nor on resistance to re-infection. Nonetheless this study provides valuable information, both new and confirmatory, regarding the development of antibodies in patients with COVID-19 and the clinical course without generally implemented treatment with cortisone or antiviral medication.

## Conclusion

There is a development of antibodies following COVID-19, and the antibody titers seem to be increased in patients with more severe disease.

## Supporting information

S1 Table Characteristics of patients with increasing vs decreasing levels of IgG from day 0 and day 28. (DOCX)
