# Supplementary material for: Antibody kinetics and clinical course of COVID-19 a prospective observational study
Source: PLoS One. 2021 Mar 22;16(3):e0248918. doi: 10.1371/journal.pone.0248918 (PMC7984607; doi:10.1371/journal.pone.0248918)
Supplement: S1 Table — (DOCX) [file pone.0248918.s001.docx]

**S1 Table**. Characteristics of patients with increasing vs decreasing levels of IgG from day 0 and day 28

| **Demographics** | **Increasing IgG levels**  *n* = 29 | **Decreasing IgG levels**  *n* = 5 |
| --- | --- | --- |
| ***Characteristic*** |  |  |
| Age, median | 58 (44-72) | 52 (48-63) |
| Sex, female *n* (%) | 17 (59) | 2 (40) |
| Onset of symptoms to COVID-19 diagnosis (d), median | 8 (2-11) | 8 (5-8) |
| Mild COVID-19 *n* (%) | 14 (48) | 2 (40) |
| Moderate COVID-19 *n* (%) | 11 (38) | 3 (60) |
| Severe COVID-19 *n* (%) | 4 (14) | 0 (0) |

For continuous variables interquartile range (IQR) is defined in the brackets.
